# The role of plant-based alternative foods in sustainable and healthy food systems: Consumption trends in the UK

**DOI:** 10.1016/j.scitotenv.2021.151041

**Published:** 2022-02-10

**Authors:** Carmelia Alae-Carew, Rosemary Green, Cristina Stewart, Brian Cook, Alan D. Dangour, Pauline F.D. Scheelbeek

**Affiliations:** a Department of Population Health, London School of Hygiene and Tropical Medicine, Keppel Street, London WC1E 7HT, UK; b Centre on Climate Change and Planetary Health, London School of Hygiene and Tropical Medicine, Keppel Street, London WC1E 7HT, UK; c Nuffield Department of Primary Care Health Services, University of Oxford, Radcliffe Observatory Quarter, Woodstock Road, Oxford OX2 6GG, UK

**Keywords:** Plant-based alternative foods, Food system transformations, Dietary trends, United Kingdom, Sustainable food systems

## Abstract

A global transformation towards sustainable food systems is crucial for delivering on climate change mitigation targets worldwide. In high- and middle-income settings, plant-based meat and dairy alternatives present potential substitutes for animal sourced foods, and a pathway to transition to more sustainable diets.

We examined plant-based alternative foods (PBAF) consumption trends in the UK by analysing repeated cross-sectional food consumption data from the National Diet and Nutrition Survey 2008–2019. Dietary data for 15,655 individuals aged 1.5 years and over were analysed to assess aggregate change in intake of PBAF and six other food groups that play a role in transformative dietary change. Characteristics associated with consumption of PBAF were explored using logistic regression, and consumption patterns in high and low meat consumers were explored by examining intake of potential animal product substitute food groups.

The proportion of individuals reporting consumption of any PBAFs increased from 6.7% in 2008–2011, to 13.1% in 2017–2019 (*p* < 0.01). Compared to 2008–2011 PBAF consumption rose by 115% in 2017–2019 (*p* < 0.01). Females were 46% more likely than males to report consumption of PBAF (*p* < 0.01). Millennials (age 24–39 years) were the most likely generation to report PBAF consumption (*p* < 0.01 compared to generation Z (age 11–23 years) and traditionalists (age 75+ years)), as were individuals of the highest income tertile (*p* < 0.01). Among “low meat consumers”, PBAF consumption was on average higher than “high meat consumers” (18.6 g versus 4.8 g PBAF per day, *p* < 0.01).

Our results support the hypothesis of a pivotal role of PBAF in the transition towards sustainable food systems in the UK, by demonstrating they are becoming increasingly popular among UK consumers. This highlights the urgent need to assess in detail the environmental and health impacts of large scale and population-wide consumption of PBAF in comparison to their animal-based equivalents.

## Highlights

• PBAF consumption has increased and appears to be accelerating in the UK.
• Contribution of PBAF to daily dietary energy intake remains small, but likely to increase in UK diets.
• Females, millennials, those of higher income report significantly higher PBAF intake.
• BPAF important “stepping stone” for dietary change.
• BPAF potentially pivotal for climate change mitigation through the food system.

## Graphical abstract

**Figure:**
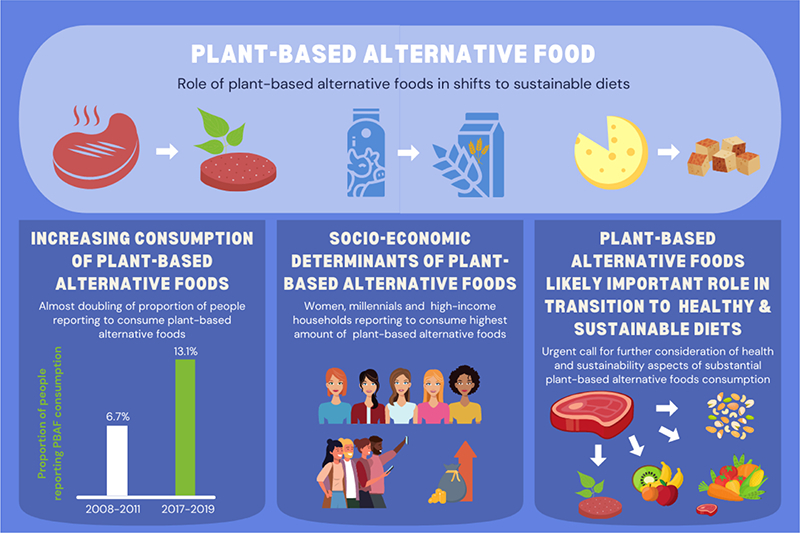

## 1 Introduction

There is now much evidence to suggest that our current global food systems and patterns of consumption are unsustainable for human and planetary health (Willett et al., 2019). The food system is responsible for roughly 21–37% of global greenhouse gas (GHG) emissions (Shukla et al., 2019), and agriculture accounts for around 70% of freshwater use globally (Food and Agriculture Organization AQUASTAT data, 2017). There are many leverage points across the food system (from farm to waste) that could bring about transformational change, but it appears unlikely that the agricultural sector will be able to meet global climate targets without concurrent substantial dietary change on the consumer side (Theurl et al., 2020). A global transition to “sustainable diets” is being widely promoted; diets typically high in plant-based and wholefoods, and low in animal sourced foods which will have co-benefits for human health and environmental sustainability (Willett et al., 2019; Springmann et al., 2016; Jarmul et al., 2020). As there is much geographic and cultural variation in diets and agricultural practices, pathways of dietary transition will vary at the national and local level (HLPE, 2017). Research has demonstrated that substituting animal products with plant-based sources of food in high- and middle-income settings can substantially reduce impacts on the environment (Clune et al., 2017; Aleksandrowicz et al., 2016; Hallström et al., 2015; Tziva et al., 2020) and reduce the incidence of and mortality from non-communicable diseases (Springmann et al., 2016; Springmann et al., 2018; Tilman and Clark, 2014).

In 2019, the UK government committed to reducing the country's net GHG emissions by 100% by 2050, relative to 1990 levels (Act, 2021). A target of 68% reduction in GHG emissions relative to 1990 levels by 2030 was subsequently announced in 2020 (Press release, 2021). As part of its recommendations for achieving a reduction in emissions, the UK Climate Change Committee (UKCCC) has suggested a 20% reduction in high‑carbon meat and dairy products by 2030, rising to a 35% reduction by 2050, with increased consumption of plant-based products (Climate Change Committee, 2019; Climate Change Committee, 2020).

Meat has been a long-standing feature of the traditional British meal, referred to as “meat and two veg” (Riley, 2010). Although there has been a growing interest in reducing meat intake in the UK, for reasons including health and concern over the environmental impacts of meat production (Eating Better, 2020a), many social and personal barriers present resistance to this dietary transition, such as social facilitation, pleasure, and beliefs about the importance of meat in the diet (Horgan et al., 2019; Macdiarmid et al., 2016). Public attitude surveys indicate that willingness to consider a reduction in meat consumption has grown from 34% in 2014 (Dibb and Fitzpatrick, 2014) to 65% of people surveyed in 2020 (Eating Better, 2020a), however the percentage of those who had reduced their meat intake in the past year was substantially lower at 21% in 2020 (Eating Better, 2020a).

Plant-based alternative foods (PBAF) present a potential solution to negotiating the changes in the familiar structure of meals and requirements for new cooking skills that would otherwise be called for in shifting diets away from animal products in the UK and countries with similar meal structures, changes that can be considered as barriers to dietary transition (Macdiarmid et al., 2016; Schösler et al., 2012). PBAF are products made from plant proteins such as soya, pea, nuts, oats and mycoproteins, designed to mimic the taste and texture of their animal-based counterparts (namely meat, milk and other dairy products). They have slowly, but steadily, increased their market share in the UK, with many of the leading supermarkets expanding their range of available products (Eating Better, 2020b; Smithers, 2020).

Although PBAF are increasingly being explored and developed as a strategy to reduce consumption of animal-sourced foods (Apostolidis and McLeay, 2016; Curtain and Grafenauer, 2019a; Michel et al., 2020; Grasso et al., 2020; McCarthy et al., 2017), the extent to which these foods play a role in dietary change remains largely understudied. There is also currently a paucity of data on what pathways people follow on their way to predominantly plant-based diets and what substitutions are most common. Should PBAF consumption be found to be accelerating in the UK, and to be playing a role in dietary transition helping to meet the UKCCC targets, intensification of studies on the healthfulness and sustainability of these products, which often undergo heavy processing (Choudhury et al., 2020) and contain salt, sugar and saturated fat, will be increasingly important. This may provide evidence to support inclusion of these products in national food-based dietary guidelines.

In order to investigate the role of plant-based alternative foods as a pathway to more sustainable diets and meeting UK meat and dairy reduction targets in the UK context, we analysed repeated cross-sectional data from the National Diet and Nutrition Survey (NDNS) to examine plant-based alternative food consumption trends and characteristics associated with consumption. We focus on alternative foods, but also explore their relative importance as compared to other potential animal product substitutes: vegetables, legumes, nuts and seeds.

## 2 Methods

### 2.1 Data source and subjects

The NDNS rolling programme is a nationally representative continuous, cross-sectional survey of the UK population (NatCen Social Research NIoHRBRCNB, 2008). Detailed dietary intake and nutritional status information is collected on an annual basis from a sample of the general population aged 1.5 and over (excluding pregnant and breastfeeding women), living in private households. The households are selected using a multi-stage random probability design based on postcode sectors. One adult and/or child per household are selected to participate in order for the sample to include equal numbers of adults and children. Daily dietary data is collected by food diaries completed by participants over 3 or 4 days, to capture all food and drink intake in and out of the home. The methods of dietary data collection and processing are detailed elsewhere (National Diet Nutrition Survey, 2008). This study included all 15,655 individuals from waves 1 to 11 spanning the time period 2008/09 to 2018/19. The data were analysed in 2 to 3-yearly groupings to make sample sizes more robust when analysing time trends: group 1, waves 1–3 (2008–2011); group 2, waves 4–6 (2011–2014); group 3, waves 7–9 (2014–2017); and group 4, waves 10–11 (2017–2019).

### 2.2 Sociodemographic characteristics

The sociodemographic variables explored were self-reported gender (female, male), generational age group at the time of survey (Pew Research Centre, 2015; McCrindle, 2019) (Generation Alpha, 1–10 years; Generation Z, 11–23 years; Millennials, 24–39 years; Generation X, 40–55 years; Baby Boomers, 56–74 years; and Traditionalists, 75 years and over), Country (England, Northern Ireland, Scotland, Wales), Ethnicity (white, black or black British, Asian or Asian British, mixed ethnic group, other) and tertiles of equivalised household income (lowest, middle, and highest tertile).

### 2.3 Average daily food group intake

Individual level food data for all subjects was categorised into nine nutritionally-relevant food group aggregates: beans & pulses, nuts & seeds, vegetables, meat (excluding seafood), milk, other dairy products (excluding milk), plant-based meat alternatives, plant-based dairy alternatives (excluding milk), and plant-based milk alternatives. PBAF were considered to be any functional analogues of animal-based foods made from plant-based ingredients (e.g. mycoprotein sausage, oat milk, tofu cheese). Pre-existing categorisation or specific subsidiary food group coding existed within the NDNS dataset for some but not all of our selected food groups (Diet and Nutrition Survey. Years 1 to 9 of the RP, 2008); where these did not exist manual assignment of food items to groups was performed using the appropriate nutrient databanks for each wave to ensure all items were captured. Where food items were ingredients in composite dishes (containing two or more ingredients), items were not included in the analysis if contributing less than 10% to the composite food. In the case of composite dishes coded as a single entry within the dataset, the grams of all food components contained within the dish were not always available, and for this reason all baked goods, desserts, confectionery and ice creams were excluded from the analysis. Similarly, where grams of components contained within composite dishes from non-excluded food categories were not available, these were not included in the total consumption calculations. Therefore milk, dairy, plant-based milk and dairy alternatives were only included if coded as individual items; where they were a component of milk-based drinks or composite dishes they were discounted. Details of foods contained within each group are provided in the supplementary material (supplementary information A).

For each individual, mean daily consumption of each food group in grams (g) was calculated by dividing the sum of grams of intake from each food group by the number of food diary days completed. Average daily dietary energy intake in kilocalories (kcal) from each food group for each individual was also calculated. Where composite dishes were coded as single entries, the dietary energy breakdown of the components contained within the dish was not available, and for the known components of the composite dish, grams of intake were converted to kcal using the energy values per gram of the constituent food items obtained from the nutrient databank. Many of the composite dishes contained more than one food item from a food group, or the primary ingredients were not able to be identified, in which case grams of intake were converted to kcal using a value derived from the average energy per gram of all listed individual food items of the respective food group contained within the nutrient databank. A list of the conversion factors is available in the supplementary material (supplementary information B). Mean daily total dietary energy intake from all foods for each individual was calculated and used to find the contribution of dietary energy from each food group to the total dietary energy intake.

### 2.4 Statistical analysis

Results were weighted to account for selection and non-response bias. Individual weights available from NDNS for each year grouping (as released) were re-scaled in order to be valid for the entire survey population. Individuals were categorised into those reporting consumption of PBAF (i.e. consumption of any foods contained within one or more of the PBAF groups: plant-based meat, milk and dairy alternatives), and no consumption. Aggregate change in the proportion of people reporting consumption of PBAF over time was explored using univariate analysis and linear regression to test for linear trend. Linear regression was used to examine aggregate change over time in mean daily consumption of selected food groups in g/capita/day and as a proportion of total daily energy intake. Sensitivity analysis was performed standardising for a 2000 kcal dietary energy intake to account for differences in age-dependent dietary energy intake. Characteristics of individuals reporting PBAF across year groupings were explored by univariate analysis. Multivariate analysis using logistic regression on all subjects across all year groupings was performed with consumption of PBAF as the dependant variable, to identify characteristics associated with consumption, adjusting for dietary energy intake. Forward step-wise selection of predictor variables to include in the model was based on likelihood ratio test (LRT) *p*-values. Consumption patterns of individuals meeting the UKCCC meat consumption target were explored by categorising subjects into “high” and “low meat consumers” (i.e. those reporting meat consumption above or below 94.3 g/capita/day for males and 66.8 g/capita/day for females). These cut-off figures were obtained from Milner et al. [currently unpublished], whose dietary modelling study quantified the changes needed to achieve a 35% reduction in meat and dairy consumption by 2050 and were used in the UKCCC Report on the UK's path to net zero (Climate Change Committee, 2020). Associations between consumption of PBAF and other selected food groups by level of meat consumption was explored using regression analysis. Intake of other food groups by PBAF consumers was explored by categorising PBAF consumers into no, low, moderate and high consumers using tertiles of average daily consumption of PBAF. All statistical analyses were performed using STATA 16.1 (StataCorp LLC, College Station, Texas, USA).

## 3 Results

### 3.1 Study characteristics

Characteristics of the study population are presented in Table 1. Of the 15,655 subjects, 54% were female, 89.6% were white and 60% lived in England. These characteristics were relatively constant across study year groupings, except for country of residence. Equivalised household income was missing 13% of values, with relatively equal distribution of missingness across year groupings; the highest proportion of missingness was among the traditionalist age group (29%), and those with Scottish nationality (17.3%) and Asian ethnicity (24%).

**Table 1:** General characteristics of the study population; aggregate change over time.

		2008–2011	2011–2014	2014–2017	2017–2019	All Years
		n (%)	n (%)	n (%)	n (%)	n (%)
Gender	Male	2275 (46.6)	2024 (45.0)	1862 (46.8)	1046 (45.4)	7207 (46.0)
	Female	2605 (53.4)	2470 (55.0)	2114 (53.2)	1259 (54.6)	8448 (54.0)
Age Group (y)	Generation Alpha (1-10)	1387 (28.4)	1204 (26.8)	1136 (28.6)	659 (28.6)	4386 (28.0)
	Generation Z (11-23)	1241 (25.4)	1087 (24.2)	890 (22.4)	500 (21.7)	3718 (23.8)
	Millennials (24-39)	657 (13.5)	588 (13.1)	517 (13.0)	324 (14.1)	2086 (13.3)
	Generation X (40-55)	722 (14.8)	729 (16.2)	651 (16.4)	361 (15.7)	2463 (15.7)
	Baby Boomers (56–74)	628 (12.9)	675 (15.0)	557 (14.0)	342 (14.8)	2202 (14.1)
	Traditionalists (75 plus)	245 (5.0)	211 (4.7)	225 (5.7)	119 (5.2)	800 (5.1)
Country	England	2547 (52.2)	2578 (57.4)	2565 (64.5)	1700 (73.8)	9390 (60.0)
	NI	731 (15.0)	509 (11.3)	578 (14.5)	358 (15.5)	2176 (13.9)
	Scotland	1117 (22.9)	741 (16.5)	210 (5.3)	166 (7.2)	2234 (14.3)
	Wales	485 (9.9)	666 (14.8)	623 (15.7)	81 (3.5)	1855 (11.9)
Ethnicity	White	4479 (91.8)	4068 (90.5)	3471 (87.3)	2008 (87.1)	14,026 (89.6)
	Mixed ethnic group	74 (1.5)	85 (1.9)	89 (2.2)	48 (2.1)	296 (1.9)
	Black or Black British	104 (2.1)	95 (2.1)	103 (2.6)	71 (3.1)	373 (2.4)
	Asian or Asian British	160 (3.3)	168 (3.7)	258 (6.5)	135 (5.9)	721 (4.6)
	Any other group	63 (1.3)	77 (1.7)	45 (1.1)	38 (1.7)	223 (1.4)
Equivalised	Lowest Tertile	1542 (31.6)	1390 (30.9)	1153 (29.0)	673 (29.2)	4758 (30.4)
Household Income	Middle Tertile	1402 (28.7)	1201 (26.7)	1069 (26.9)	659 (28.6)	4331 (27.7)
	Highest Tertile	1342 (27.5)	1295 (28.8)	1237 (31.1)	656 (28.5)	4530 (28.9)
	Missing	594 (12.2)	608 (13.5)	517 (13.0)	317 (13.8)	2036 (13.0)

y, year; NI, Northern Ireland.

### 3.2 Trends in consumption of plant-based alternative foods and other selected food groups

**Fig. 1:**
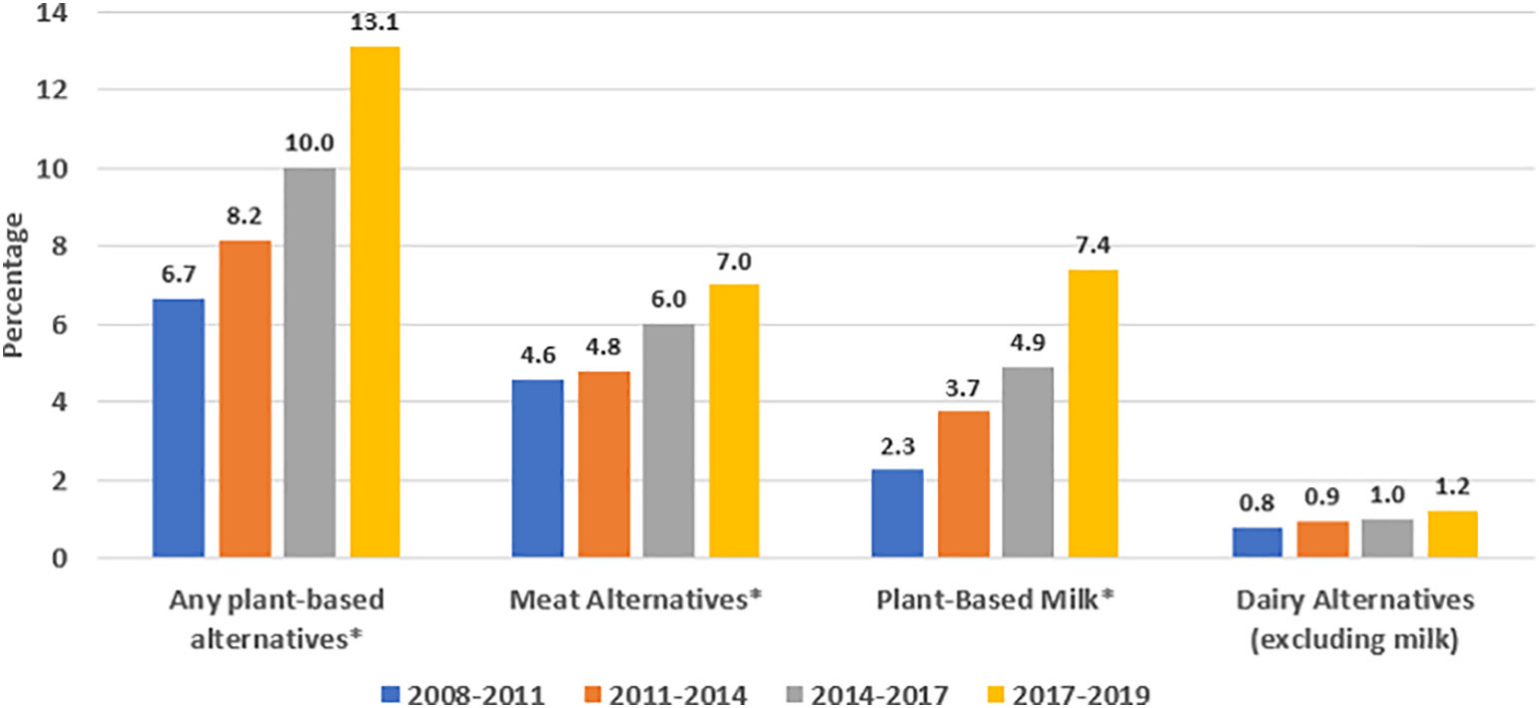
Trends in proportion of people reporting consumption of plant-based alternative foods (%); aggregate change over time. *indicates *p* test for trend value <0.01.

**Fig. 2:**
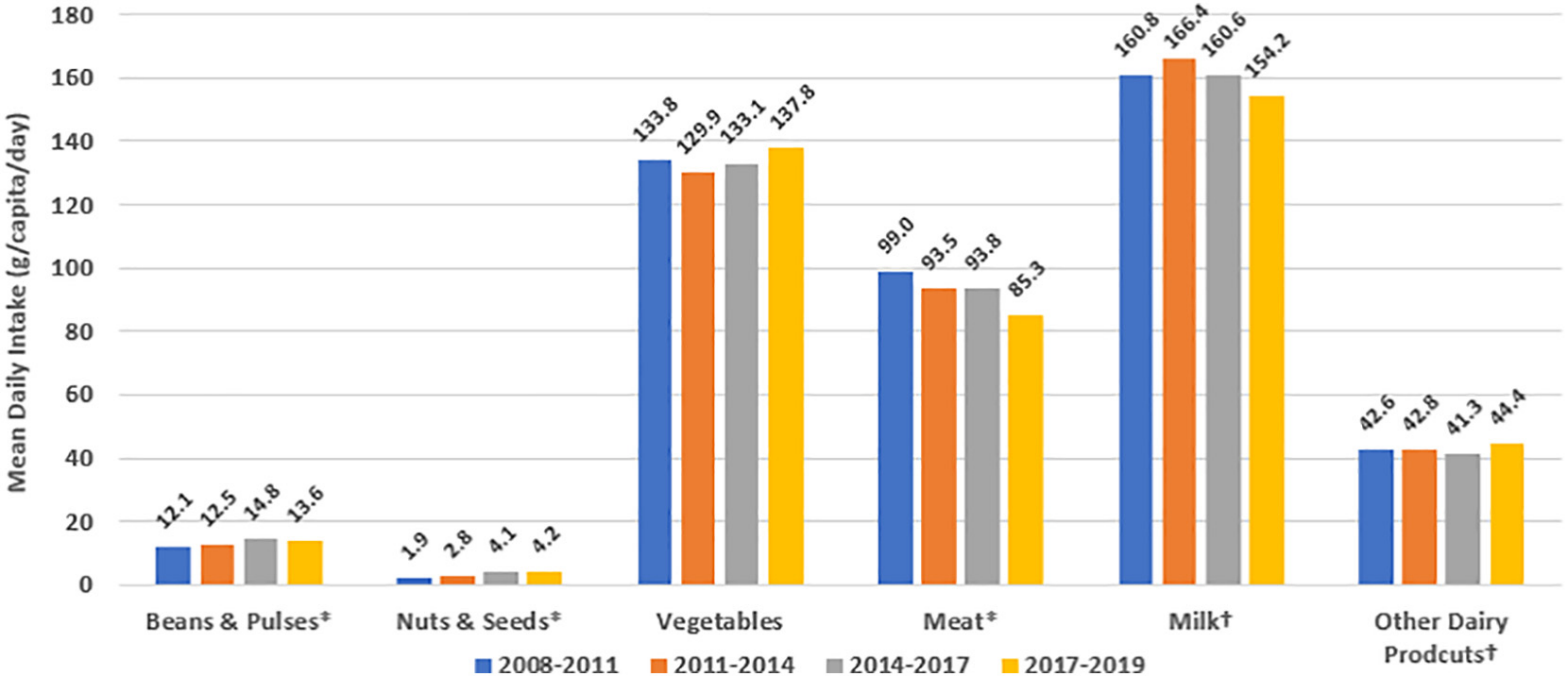
Trends in mean daily reported consumption of selected food groups from 2008 to 2011 to 2017–2019, in grams/capita/day; aggregate change over time. *indicates *p*-test for trend <0.01. †Excludes dairy products used in baked goods, confectionery and desserts, and yogurt, cream and milk used in composite dishes.

**Fig. 3:**
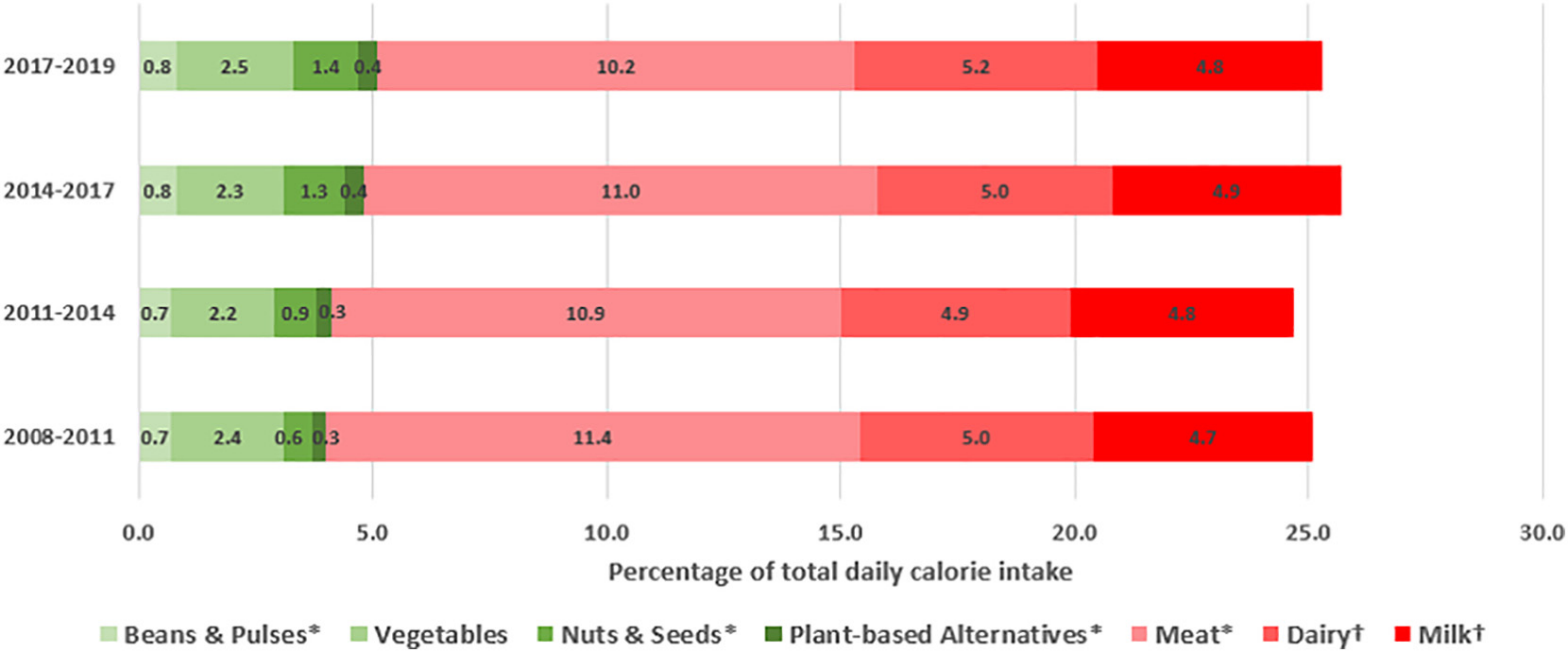
Trends in mean daily intake of selected food groups as a proportion of total daily dietary energy intake from 2008 to 2011 to 2017–2019; aggregate change over time. *indicates *p*-test for trend <0.01. †Excludes dairy products used in baked goods, confectionery and desserts, and yogurt, cream and milk used in composite dishes.

Overall, the proportion of individuals reporting consumption of any PBAF increased: from 6.7% in 2008–2011, to 13.1% in 2017–2019 (*p*_trend_ < 0.01), with reported plant-based meat alternative consumption increasing from 4.6% to 7.0% (*p*_trend_ < 0.01), and plant-based milk consumption increasing from 2.3% to 7.4% (*p*_trend_ < 0.01) (Fig. 1). Similarly, over this period mean daily consumption of beans & pulses, and nuts & seeds significantly increased (*p*_trend_ < 0.01), and intake of meat significantly decreased (99.0 to 85.3 g/capita/day, *p*_trend_ < 0.01) (Fig. 2). As shown in Fig. 3, the contribution of beans & pulses, and nuts & seeds to total daily dietary energy intake also increased over this period from 1.3% to 2.2% (*p*_trend_ < 0.01). The contribution of PBAF towards total calorie intake increased from 0.3% in 2008–2011 to 0.4% in 2017–2019 (*p*_trend_ < 0.01). Sensitivity analysis standardising consumption for a 2000 kcal daily energy intake inflated mean daily consumption figures; the pattern of intake trend across the year groupings was largely unchanged although the trend was no longer significant in the beans & pulses food group. A significant decrease in mean consumption of vegetables and milk was demonstrated in the standardised results (supplementary information C).

### 3.3 Characteristics associated with consumption of plant-based alternative foods

Univariate analysis demonstrated the majority of subjects reporting PBAF consumption were female (10.7% of females vs 7.5% of males, across all years), although the proportion of both males and females eating these foods increased over time (females 8.2% in 2008–2011, 15.4% in 2017–2019; males 5.1% in 2008–2011, 10.7% in 2017–2019) (Table 2). Millennials (ages 24–39) and Generation X (ages 40–55) were proportionally the largest reported consumers of PBAF (10.6% and 10.8% respectively, across all years), although there was a large increase in the proportion of Generation Z (ages 11–23) reporting consumption of these foods over time, from 3.9% in 2008–2011 to 13.3% in 2017–2019. Reported consumption of PBAF was highest among the highest tertile of equivalised household income (11.9% highest tertile vs 6.8% lowest tertile, across all years), and the largest increase over time was also seen in the highest income tertile group (8.8% in 2008–2011, 17.8% in 2017–2019).

**Table 2:** Characteristics of plant-based alternatives consumers; aggregate change over time.

		2008–2011	2011–2014	2014–2017	2017–2019	All Years
		n (weighted %)	n (weighted %)	n (weighted %)	n (weighted %)	n (%)
Gender	Male	100 (5.1)	126 (6.3)	152 (9.1)	103 (10.7)	481 (7.5)
	Female	179 (8.2)	222 (10.0)	199 (10.9)	162 (15.4)	762 (10.7)
Age group (y)	Generation Alpha (1-10)	78 (5.9)	100 (8.1)	92 (8.0)	74 (11.8)	344 (8.2)
	Generation Z (11-23)	45 (3.9)	62 (6.2)	73 (11.7)	50 (13.3)	230 (8.1)
	Millennials (24-39)	46 (8.4)	60 (7.3)	62 (12.5)	42 (15.6)	210 (10.6)
	Generation X (40-55)	58 (8.4)	70 (11.0)	69 (11.0)	50 (13.9)	247 (10.8)
	Baby Boomers (56–74)	40 (5.3)	46 (7.9)	42 (7.8)	43 (12.6)	171 (8.1)
	Traditionalists (75 plus)	12 (7.6)	10 (6.6)	13 (5.2)	6 (6.0)	41 (6.4)
Country	England	171 (7.2)	227 (8.4)	257 (10.4)	210 (10.4)	865 (9.6)
	NI	32 (5.0)	22 (3.1)	24 (3.7)	26 (3.7)	104 (4.7)
	Scotland	56 (4.5)	55 (8.5)	19 (7.9)	22 (7.9)	152 (7.5)
	Wales	20 (3.1)	44 (6.7)	51 (10.5)	7 (10.5)	122 (7.5)
Ethnicity	White	236 (6.4)	294 (7.8)	304 (10.2)	229 (13.1)	1066 (9.0)
	Mixed ethnic group	12 (13.1)	14 (16.2)	10 (8.9)	8 (23.2)	44 (14.6)
	Black or Black British	12 (11.1)	9 (9.8)	8 (7.8)	8 (19.2)	37 (11.5)
	Asian or Asian British	11 (4.8)	18 (10.1)	21 (7.6)	12 (7.2)	62 (7.5)
	Any other group	8 (11.1)	10 (8.3)	7 (19.5)	8 (17.7)	33 (13.1)
Equivalised Household	Lowest Tertile	58 (4.0)	74 (6.5)	75 (7.7)	65 (10.7)	272 (6.8)
Income	Middle Tertile	83 (6.2)	98 (8.4)	92 (8.0)	60 (9.4)	333 (7.9)
	Highest Tertile	93 (8.8)	127 (9.6)	143 (13.2)	104 (17.8)	467 (11.9)
	Missing	45 (8.4)	49 (7.9)	41 (9.8)	36 (14.7)	171 (9.9)

As shown in Table 3, multivariate analysis demonstrated females were 46% more likely than males to report consumption of PBAF (*p* < 0.01). Compared to millennials, generation Z and traditionalists (ages 75 and over) were less likely to report PBAF consumption (*p* < 0.01), and living in a region other than England reduced the odds of reporting consumption (Northern Ireland *p* < 0.01; Wales *p* = 0.02). Reporting PBAF consumption increased with increasing income tertile (*p* < 0.01), and compared to 2008–2011 PBAF consumption rose by 38% in 2011–2014, 64% in 2014–2017 and 115% in 2017–2019 (*p* < 0.01).

**Table 3:** Characteristics associated with consumption of plant-based alternative foods, adjusted for energy intake.

		OR	p value
Gender	Male		
	Female	**1**.**46** (**1**.**27–1**.**67**)	**<0**.**01**
Age Group (y)	Millennials (24-39)		
	Generation Alpha (1-10)	0.87 (0.70–1.06)	0.17
	Generation Z (11-23)	**0**.**67** (**0**.**54–0**.**83**)	**<0**.**01**
	Generation X (40-55)	0.96 (0.78–1.19)	0.76
	Baby Boomers (56–74)	0.80 (0.64–1.02)	0.07
	Traditionalists (75 plus)	**0**.**58** (**0**.**39–0**.**87**)	**<0**.**01**
Country	England		
	NI	**0**.**55** (**0**.**43–0**.**68**)	**<0**.**01**
	Scotland	0.83 (0.68–1.02)	0.08
	Wales	**0**.**77** (**0**.**62–0**.**95**)	**0**.**02**
Ethnic Group	White		
	Mixed ethnic group	**2**.**04** (**1**.**43–2**.**89**)	**<0**.**01**
	Black or Black British	1.10 (0.74–1.65)	0.63
	Asian or Asian British	0.95 (0.69–1.32)	0.77
	Any other group	**1**.**97** (**1**.**30–2**.**98**)	**<0**.**01**
Equivalised Household Income	Lowest Tertile		
	Middle Tertile	**1**.**36** (**1**.**15–1**.**61**)	**<0**.**01**
	Highest Tertile	**1**.**76** (**1**.**50–2**.**06**)	**<0**.**01**
Group	2008–2011		
	2011–2014	**1**.**38** (**1**.**16–1**.**65**)	**<0**.**01**
	2014–2017	**1**.**64** (**1**.**37–1**.**97**)	**<0**.**01**
	2017–2019	**2**.**15** (**1**.**77–2**.**62**)	**<0**.**01**

NI, Northern Ireland.

Bold font = p-value <0.05.

### 3.4 Mean consumption of selected food groups by low and high meat eaters

**Fig. 4:**
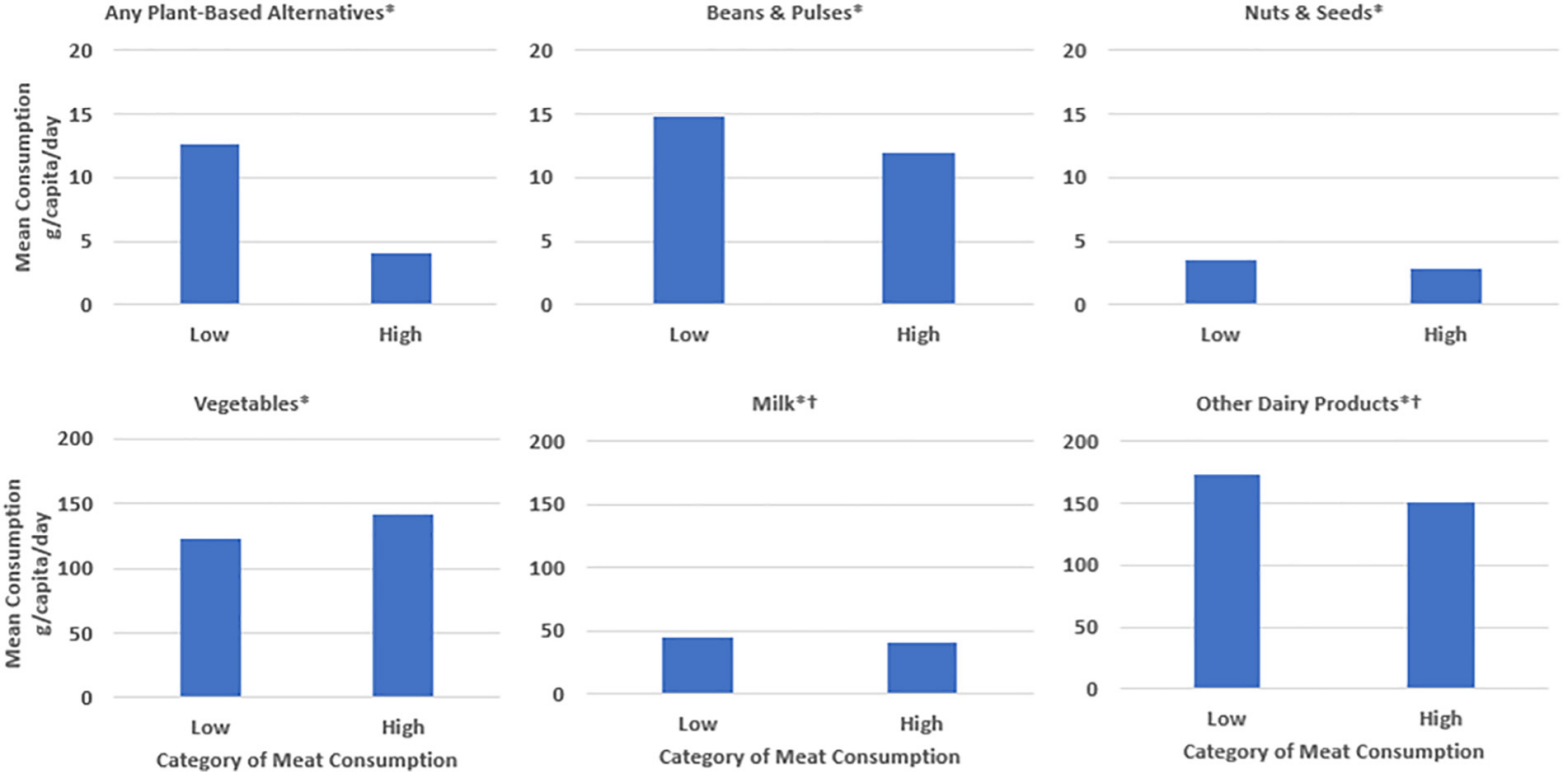
Mean daily consumption of selected food groups by category of meat consumer (g/capita/day). Meat consumers defined by meeting UKCCC's 2050 meat consumption target of 94.3 g/capita/day for males, and 66.8 g/capita/day for females (low meat consumption), or eating above this target amount (high meat consumption). *indicates *p* value <0.01. †Excludes dairy products used in baked goods, confectionery and desserts, and yogurt, cream and milk used in composite dishes.

Observing the UKCCC recommendations of a maximum of 94.3 g and 66.8 g meat per day for males and females respectively, the dataset contained 7871 (54.85%) “low meat consumers” i.e. those reporting a meat consumption below this threshold, and 7784 (45.15%) “high meat consumers”. Mean intake of PBAF among “low meat consumers” was 8.6 g/capita/day higher than “high meat consumers” (Low meat consumers = 12.7 g/capita/day; High meat consumers = 4.1 g/capita/day; *p* < 0.01) (Fig. 4). Low meat consumers also reported a higher mean consumption of beans & pulses (*p* < 0.01), nuts & seeds (*p* < 0.01), milk (*p* < 0.01), and other dairy products (*p* < 0.01). A similar pattern was seen when looking at food group consumption by category of PBAF consumer (supplementary information D), with reported consumption of beans & pulses and nuts & seeds significantly higher among PBAF consumers compared to non PBAF consumers (*p*_trend_ < 0.01). However, milk intake was substantially lower among the high PBAF consumers compared to those consuming none or a low amount of these foods (*p*_trend_ < 0.01).

## 4 Discussion

Our analysis demonstrates that the consumption of PBAF, while still relatively small as a percentage of daily dietary energy intake, has increased significantly over the period 2008–2019 in the UK and appears to be accelerating. We demonstrate that PBAF consumption is higher among females, millennials (i.e. individuals aged 24–39 years), and those of the highest income tertile, suggesting different barriers to access of PBAF between various societal groups. Our results indicate that reported consumption of PBAF and other plant-based food groups is higher among those who eat less meat, supporting the hypothesis that these products have a role in the transition of UK diets away from animal products. We highlight that there may be trade-offs between reduced meat and increased dairy product consumption, in line with the findings of other UK dietary studies (Bradbury et al., 2017; Key et al., 2009); but our findings also suggest that PBAF may be considered by consumers as a valid substitution for both.

The popularity of PBAF is growing in the UK, supported by market data demonstrating recent boosts in sales of meat substitutes and plant-based milk (Mintel Press Team, 2020; Mintel Press Team, 2019a), and the country is leading in the development and launching of new vegan products (Mintel Press Team, 2019b). Increasing popularity of PBAF is also reported in studies from other countries especially those with predominantly “Western diets”, often driven by change in demand due to environmental concerns. In Sydney (Australia), for example, the number of various PBAF on offer in four major supermarket chains was found to have expanded exponentially from 26 products in 2015 to 137 in 2019, including over 50 different types of plant-based burgers (Curtain and Grafenauer, 2019b). Furthermore, epidemics including the 2003 SARS outbreak, the 2013 Avian Influenza outbreak, the 2019 African Swine Flu outbreak and the 2020 COVID-19 pandemic accelerated shifts away from (ultra-processed) animal sourced foods, especially in Asia, which was severely affected by all epidemics (Attwood and Hajat, 2020). Underlying reasons were related to food scares of affected foods, but also related to more general health and dietary concerns as poor diets were often related to higher risk of severe disease presentation (Attwood and Hajat, 2020).

Many PBAFs form an easy-to-apply ‘like-for-like’ substitution for animal sourced foods and could therefore be a suitable solution for those wishing to eat more plant-based in a “hassle free” way. It is thus becoming increasingly necessary to obtain further clarification on the health and environmental impacts of these foods, as well as the wider political, industry and agricultural implications of their increased production and consumption. Emerging research into the environmental impacts of plant-based meat alternatives in comparison to their animal counterparts has shown promising results for GHG emissions, land use and blue water footprint (Santo et al., 2020) and suggest that PBAF could play a pivotal role in climate change mitigation through the food system. The health implications of diets high in PBAF (i.e. processed functional analogues) are less well known, although one recent randomized crossover trial found that in comparison to animal meats, plant-based meat alternatives were associated with lower weight and lower LDL-cholesterol (Crimarco et al., 2020). In contrast to the paucity of evidence relating to plant-based alternative products, there is an abundance of studies, including meta-analyses, that demonstrate the positive effect of diets high in unprocessed plant-based foods on health outcomes such as lipids (Guasch-Ferré et al., 2019; Schwingshackl et al., 2018), type 2 diabetes (Qian et al., 2019), as well as cardiovascular (Song et al., 2016; Budhathoki et al., 2019) and all-cause mortality (Song et al., 2016; Budhathoki et al., 2019; Schwingshackl et al., 2017). What remains to be seen is how the sustainability of PBAF compares to their wholefood primary ingredients, and whether a shift towards these substitute foods will be enough to remain within planetary boundaries. Furthermore, it is yet to be determined whether PBAF will remain as a permanent feature in people's diets, or whether this is rather a stepping-stone towards diets higher in minimally or unprocessed plant-based foods.

Further assessment of the impact of PBAF on the nutritional quality of the diet is also needed. Recent modelling studies investigating the effect of substituting meat with meat alternatives (Farsi et al., 2021), and meat, milk and dairy desserts with PBAF (Salomé et al., 2021) on nutritional adequacy have highlighted a number of nutritional issues for further consideration. Notably, intakes of certain nutrients such as vitamin B12 (Farsi et al., 2021) and iodine (Salomé et al., 2021) were less adequate among the simulated substitution diets. Similar nutritional issues were also noted in studies of individuals following vegan and plant-based diets, particularly among individuals not taking supplements (Desmond et al., 2021; Pawlak et al., 2014; Groufh-Jacobsen et al., 2020). Additionally, substituting meat with plant-based alternatives resulted in higher salt and sugar intake and increased consumption of ultra-processed foods (UPF) as a proportion of total dietary energy intake, although results differed depending on the formulation and plant food type of the substitute product (Farsi et al., 2021; Salomé et al., 2021). The current boom in the plant-based alternative foods market, driven by new technologies and innovations, has allowed for numerous new products with a broad range of nutrient compositions and level of processing to be introduced to consumers. The high level of processing undergone by some of the available PBAF products may class them as UPFs, consumption of which is associated with a number of negative health outcomes (Lane et al., 2021). On the other hand, metabolomic profiling analysis comparing one plant-based beef substitute to grass-fed beef has demonstrated the complexity in trying to compare and understand nutritional adequacy of PBAF, as both foods assessed both contained and lacked differing nutrients with potential benefits to health (van Vliet et al., 2021). Should PBAF become a substantial feature of the UK diet it will be important to ensure that these foods do not introduce new or potentiate existing risks to nutritional health; consideration may need to be given to their formulation and whether fortification with micronutrients should be encouraged, and to their regulation – i.e. to what standards they are held to and by who, and whether they will fall into the category of foods high in fat, sugar and salt (HFSS) and be subject to the same promotion restrictions as other foods.

With further investigation and clarification of the environmental and health implications of PBAF, these animal-product substitutes have the potential to become a vehicle for dietary change, an accessible and relatable option for those wishing to take steps towards a more sustainable diet, and therewith an important consumer-side leverage point for food system transformations towards sustainable food systems. Our findings of greater intake of PBAF among low meat eaters, and low consumption of meat and dairy products among high PBAF consumers, suggests that plant-based products are often used as direct substitutes for animal-sourced products, and are likely to play an important role in meeting the UKCCCs 2050 animal sourced foods reduction target. Should the emerging research into the broader implications of PBAF reflect positively on these products, their inclusion in national food-based dietary guidelines may warrant consideration, presented among the options of alternative protein sources to meat and dairy products. The UK is among several countries where the potential remains for the environmental sustainability of its national food-based dietary guidelines to be increased (Springmann et al., 2020), and inclusion of PBAF may prove beneficial in achieving this, in addition to aiding the attainment of the UK's emissions target. Investigation into how the consumption of PBAF in the UK compares to other nations, and whether their promotion is an appropriate and popular strategy in other settings, will help to clarify whether these products can also contribute to a global reduction in emissions.

Our results highlight the demographics of PBAF consumers in the UK, and indicate where opportunities may exist to target strategies for reduced animal-sourced food consumption, should the promotion of these substitute foods be supported by the emerging evidence. Our finding that more females than males consume PBAF is in keeping with results from market research in the UK in 2016, showing 63% of vegans were female and 37% male (The Vegan Society, 2016). Women are more likely to report responsibility for the food shopping and preparation than men (Flagg et al., 2014), who are more likely to eat food out of the home (Naska et al., 2015) and consume fast food more frequently than women (Garza et al., 2016). The recent rise in plant-based food outlets and product availability in fast food stores may have implications for uptake in men and increase accessibility to lower income households who have higher exposure to fast food outlets in the UK (Burgoine et al., 2018), narrowing the consumption gap between lower and higher income families highlighted here, however this should not distract from continued efforts to remove existing barriers to affordable healthy wholefoods in the UK (Barton et al., 2015; Corfe, 2018). As the question of whether PBAF can be part of a healthy and sustainable diet continues to be explored (Hu et al., 2019), increasing access to options other than meat through exposure to meat alternatives in food outlets including fast food settings could serve to further the conversation surrounding dietary change in previously under-reached demographic groups.

To our knowledge, this is the first analysis of PBAF consumption trends in the UK, and our findings add to the expanding knowledge base of UK dietary trends. As we used a nationally representative dataset with selection and non-response weights applied, our results are likely generalisable to the entire UK population. Furthermore, methods used could be replicated in other settings where representative dietary survey data is available. There are some limitations to be noted however. Firstly, while food diaries offer an advantage of accuracy over recall dietary assessment methods, we acknowledge the issue of under-reporting in their use (Ortega et al., 2015; Hu et al., 2020). Secondly, data analysis limitations resulted in exclusion of many food items such as desserts, baked goods and snacks; therefore, consumption of some food groups, particularly dairy products and milk, is under-estimated. Similarly, as a consequence of not having calorie breakdown information for composite dishes there may be some inaccuracies in the energy intake calculations. We attempted to address this using manual calculation where possible, or the use of food group averages, however we recognise some error may have persisted. While our quantitative estimates of the diet may have inaccuracies, the aim of our study was not to quantify absolute intakes of all food groups, rather our results still provide interesting and relevant information on trends. Lastly, our trends presented were aggregate change over time, and we were unable to explore individual change over time as would be possible with time-series analyses.

In conclusion, our study supports further exploration of PBAF as one of a number of possible pathways to dietary transition and progress towards meeting emissions targets in high-income settings such as in the UK. Future studies using time series analysis both in the UK and other settings would be prudent to examine barriers to and enablers of dietary substitutions at the local, regional and global level in further detail, and research focussing on the health and environmental impacts of PBAF will assist in directing the evidence-based narrative of these foods in a planetary health context. PBAF are likely to become increasingly popular and more widely consumed, but given the high level of processing many of these products undergo, acting now to regulate their nutritional content may be prudent to prevent substitution of one public health concern for another. Consideration to the wider political, economic, agricultural and food systems implications for the UK, its trading partners, and the rest of the world must also be given should the role of these foods in helping shape the national and the global shift towards more sustainable diets become increasingly important.

## Funding

This work was supported by the 10.13039/100010269Wellcome Trust's Our Planet, Our Health programme [grant number 205200/Z/16/Z].

## CRediT authorship contribution statement

**Carmelia Alae-Carew:** Conceptualization, Methodology, Formal analysis, Writing – original draft, Visualization. **Rosemary Green:** Conceptualization, Methodology, Writing – review & editing. **Cristina Stewart:** Methodology, Writing – review & editing. **Brian Cook:** Writing – review & editing. **Alan D. Dangour:** Writing – review & editing, Supervision. **Pauline F.D. Scheelbeek:** Conceptualization, Methodology, Writing – review & editing, Visualization, Supervision.

## Declaration of competing interest

The authors declare that they have no known competing financial interests or personal relationships that could have appeared to influence the work reported in this paper.

## Appendix A Supplementary data

Supplementary material Image 1

## References

[bb0005] (2021). https://www.legislation.gov.uk/uksi/2019/1056/contents/made.

[bb0010] Aleksandrowicz L., Green R., EJM Joy, Smith P., Haines A. (2016). The impacts of dietary change on greenhouse gas emissions, land use, water use, and health: a systematic review. PloS one.

[bb0015] Apostolidis C., McLeay F. (2016). Should we stop meating like this? Reducing meat consumption through substitution. Food Policy.

[bb0020] Attwood S., Hajat C. (2020). How will the COVID-19 pandemic shape the future of meat consumption?. Public Health Nutr..

[bb0025] Barton K.L., Wrieden W.L., Sherriff A., Armstrong J., Anderson A.S. (2015). Trends in socio-economic inequalities in the scottish diet: 2001–2009. Public Health Nutr..

[bb0030] Bradbury K.E., Tong T.Y., Key T.J. (2017). Dietary intake of high-protein foods and other major foods in meat-eaters, poultry-eaters, fish-eaters, vegetarians, and vegans in UK Biobank. Nutrients.

[bb0035] Budhathoki S., Sawada N., Iwasaki M., Yamaji T., Goto A., Kotemori A. (2019). Association of animal and plant protein intake with all-cause and cause-specific mortality in a Japanese cohort. JAMA Intern. Med..

[bb0040] Burgoine T., Sarkar C., Webster C.J., Monsivais P. (2018). Examining the interaction of fast-food outlet exposure and income on diet and obesity: evidence from 51,361 UK Biobank participants. Int. J. Behav. Nutr. Phys. Act..

[bb0045] Choudhury D., Singh S., Seah J.S.H., Yeo D.C.L., Tan L.P. (2020). Commercialization of plant-based meat alternatives. Trends Plant Sci..

[bb0050] Climate Change Committee (2019).

[bb0055] Climate Change Committee (2020).

[bb0060] Clune S., Crossin E., Verghese K. (2017). Systematic review of greenhouse gas emissions for different fresh food categories. J. Clean. Prod..

[bb0065] Corfe S.J.S.M.F. (2018). http://www.smf.co.uk/wp-content/uploads/2018/10/What-are-the-barriers-to-eating-healthy-in-the-UK.pdf.

[bb0070] Crimarco A., Springfield S., Petlura C., Streaty T., Cunanan K., Lee J. (2020). A randomized crossover trial on the effect of plant-based compared with animal-based meat on trimethylamine-N-oxide and cardiovascular disease risk factors in generally healthy adults: study with appetizing plantfood—meat eating alternative trial (SWAP-MEAT). Am. J. Clin. Nutr..

[bb0075] Curtain F., Grafenauer S. (2019). Plant-based meat substitutes in the flexitarian age: an audit of products on supermarket shelves. Nutrients.

[bb0080] Curtain F., Grafenauer S. (2019). Plant-based meat substitutes in the flexitarian age: an audit of products on supermarket shelves. Nutrients.

[bb0085] Desmond M.A., Sobiecki J.G., Jaworski M., Płudowski P., Antoniewicz J., Shirley M.K. (2021). Growth, body composition, and cardiovascular and nutritional risk of 5-to 10-y-old children consuming vegetarian, vegan, or omnivore diets. Am. J. Clin. Nutr..

[bb0090] Dibb S., Fitzpatrick I. (2014). https://www.eating-better.org/uploads/Documents/LetsTalkAboutMeat.pdf#:~:text=To%20stimulate%20long-term%20cultural%20shifts%20by%20devising%20new,Fitzpatrick%20is%20published%20by%20Eating%20Better%2C%20December%202014.

[bb0095] National Diet and Nutrition Survey. Years 1 to 9 of the RP (2008/2009 – 2016/2017). Appendix R: Main and subsidiary food groups and disaggregation categories.

[bb0100] Eating Better (2020). https://www.eating-better.org/uploads/Documents/2020/Eating%20Better%202020%20public%20survey%20analysis.docx.pdf.

[bb0105] Eating Better (2020). 2019-20 Impact report: build back better. https://www.eating-better.org/uploads/Documents/2020/EB-impact-report-19-20-FINAL.pdf.

[bb0110] Farsi D.N., Uthumange D., Munoz J.M., Commane D.M. (2021). The nutritional impact of replacing dietary meat with meat alternatives in the UK: a modelling analysis using nationally representative data. Br. J. Nutr..

[bb0115] Flagg L.A., Sen B., Kilgore M., Locher J.L. (2014). The influence of gender, age, education and household size on meal preparation and food shopping responsibilities. Public Health Nutr..

[bb0120] Food and Agriculture Organization AQUASTAT data (2017). Annual freshwater withdrawals, agriculture (% of total freshwater withdrawal). https://data.worldbank.org/indicator/er.h2o.fwag.zs.

[bb0125] Garza K.B., Ding M., Owensby J.K., Zizza C.A. (2016). Impulsivity and fast-food consumption: a cross-sectional study among working adults. J. Acad. Nutr. Diet..

[bb0130] Grasso N., Alonso-Miravalles L., O’Mahony J.A. (2020). Composition, physicochemical and sensorial properties of commercial plant-based yogurts. Foods.

[bb0135] Groufh-Jacobsen S., Hess S.Y., Aakre I., Folven Gjengedal E.L., Blandhoel Pettersen K., Henjum S. (2020). Vegans, vegetarians and pescatarians are at risk of iodine deficiency in Norway. Nutrients.

[bb0140] Guasch-Ferré M., Satija A., Blondin S.A., Janiszewski M., Emlen E., O’Connor L.E. (2019). Meta-analysis of randomized controlled trials of red meat consumption in comparison with various comparison diets on cardiovascular risk factors. Circulation.

[bb0145] Hallström E., Carlsson-Kanyama A., Börjesson P. (2015). Environmental impact of dietary change: a systematic review. J. Clean. Prod..

[bb0150] HLPE (2017).

[bb0155] Horgan G.W., Scalco A., Craig T., Whybrow S., Macdiarmid J. (2019). Social, temporal and situational influences on meat consumption in the UK population. Appetite.

[bb0160] Hu F.B., Otis B.O., McCarthy G. (2019). Can plant-based meat alternatives be part of a healthy and sustainable diet?. JAMA.

[bb0165] Hu M., Kirlin J.A., West B.T., He W., Ong A.R., Zhang S., Zhang X. (2020). Estimation of underreporting in diary surveys: an application using the National Household Food Acquisition and purchase survey. J.Survey Stat.Methodol..

[bb0170] Jarmul S., Dangour A.D., Green R., Liew Z., Haines A., Scheelbeek P.F.D. (2020). Climate change mitigation through dietary change: a systematic review of empirical and modelling studies on the environmental footprints and health effects of ‘sustainable diets’. Environ. Res. Lett..

[bb0175] Key T.J., Appleby P.N., Spencer E.A., Travis R.C., Roddam A.W., Allen N.E. (2009). Mortality in British vegetarians: results from the European prospective investigation into cancer and nutrition (EPIC-Oxford). Am. J. Clin. Nutr..

[bb0180] Lane M.M., Davis J.A., Beattie S., Gómez-Donoso C., Loughman A., O'Neil A. (2021). Ultraprocessed food and chronic noncommunicable diseases: a systematic review and meta-analysis of 43 observational studies. Obes. Rev..

[bb0185] Macdiarmid J.I., Douglas F., Campbell J. (2016). Eating like there's no tomorrow: public awareness of the environmental impact of food and reluctance to eat less meat as part of a sustainable diet. Appetite.

[bb0190] McCarthy K., Parker M., Ameerally A., Drake S., Drake M. (2017). Drivers of choice for fluid milk versus plant-based alternatives: what are consumer perceptions of fluid milk?. J. Dairy Sci..

[bb0195] McCrindle (2019). https://mccrindle.com.au/insights/blogarchive/gen-z-and-gen-alpha-infographic-update/.

[bb0200] Michel F., Hartmann C., Siegrist M. (2020). Consumers' associations, perceptions and acceptance of meat and plant-based meat alternatives. Food Qual. Prefer..

[bb0205] Mintel Press Team (2019). Milking the vegan trend: a quarter (23%) of Brits use plant-based milk. https://www.mintel.com/press-centre/food-and-drink/milking-the-vegan-trend-a-quarter-23-of-brits-use-plant-based-milk.

[bb0210] Mintel Press Team (2019). #Veganuary: UK overtakes Germany as world's leader for vegan food launches. https://www.mintel.com/press-centre/food-and-drink/veganuary-uk-overtakes-germany-as-worlds-leader-for-vegan-food-launches.

[bb0215] Mintel Press Team (2020). Plant-based push: UK sales of meat-free foods shoot up 40% between 2014-19. https://www.mintel.com/press-centre/food-and-drink/plant-based-push-uk-sales-of-meat-free-foods-shoot-up-40-between-2014-19.

[bb0220] Naska A., Katsoulis M., Orfanos P., Lachat C., Gedrich K., Rodrigues S.S. (2015). Eating out is different from eating at home among individuals who occasionally eat out. A cross-sectional study among middle-aged adults from eleven european countries. Br. J. Nutr..

[bb0225] NatCen Social Research NIoHRBRCNB, Diet, Anthropometry and Physical Activity Group, University of Cambridge, MRC Epidemiology Unit, Nutritional Biomarker Laboratory.,. National Diet and Nutrition Survey Years 1-11, 2008-2019. UK Data Service. 2021.

[bb0230] National Diet Nutrition Survey Results from Years 1-4 (combined) of the Rolling Programme (20088/2009 - 2011/12). Appendix A: Dietary collection and editing.

[bb0235] Ortega R.M., Pérez-Rodrigo C., López-Sobaler A.M. (2015). Dietary assessment methods: dietary records. Nutr. Hosp..

[bb0240] Pawlak R., Lester S., Babatunde T. (2014). The prevalence of cobalamin deficiency among vegetarians assessed by serum vitamin B12: a review of literature. Eur. J. Clin. Nutr..

[bb0245] Pew Research Centre (2015). https://www.pewresearch.org/politics/2015/09/03/the-whys-and-hows-of-generations-research/#:~:text=Defining%20Generations%20The%20Pew%20Research%20Center%E2%80%99s%20approach%20to,generations%20is%20a%20necessary%20step%20for%20this%20analysis.

[bb0250] Press release: UK sets ambitious new climate target ahead of UN Summit. https://www.gov.uk/government/news/uk-sets-ambitious-new-climate-target-ahead-of-un-summit.

[bb0255] Qian F., Liu G., Hu F.B., Bhupathiraju S.N., Sun Q. (2019). Association between plant-based dietary patterns and risk of type 2 diabetes: a systematic review and meta-analysis. JAMA Intern. Med..

[bb0260] Riley H. (2010). Potato consumption in the UK-why is' meat and two veg'no longer the traditional British meal?. Nutr. Bull..

[bb0265] Salomé M., Huneau J.-F., Le Baron C., Kesse-Guyot E., Fouillet H., Mariotti F. (2021). Substituting meat or dairy products with plant-based substitutes has small and heterogeneous effects on diet quality and nutrient security: a simulation study in french adults (INCA3). J. Nutr..

[bb0270] Santo R.E., Kim B.F., Goldman S.E., Dutkiewicz J., Biehl E., Bloem M.W. (2020). Considering plant-based meat substitutes and cell-based meats: a public health and food systems perspective. Front.Sustain.Food Syst..

[bb0275] Schösler H., De Boer J., Boersema J.J. (2012). Can we cut out the meat of the dish? Constructing consumer-oriented pathways towards meat substitution. Appetite.

[bb0280] Schwingshackl L., Schwedhelm C., Hoffmann G., Lampousi A.-M., Knüppel S., Iqbal K. (2017). Food groups and risk of all-cause mortality: a systematic review and meta-analysis of prospective studies. Am. J. Clin. Nutr..

[bb0285] Schwingshackl L., Hoffmann G., Iqbal K., Schwedhelm C., Boeing H. (2018). Food groups and intermediate disease markers: a systematic review and network meta-analysis of randomized trials. Am. J. Clin. Nutr..

[bb0290] Shukla P., Skea J., Calvo Buendia E., Masson-Delmotte V., Pörtner H., Roberts D. (2019).

[bb0295] Smithers R. (2020). https://www.theguardian.com/lifeandstyle/2020/jul/25/uk-demand-for-new-vegan-food-products-soars-in-lockdown.

[bb0300] Song M., Fung T.T., Hu F.B., Willett W.C., Longo V.D., Chan A.T. (2016). Association of animal and plant protein intake with all-cause and cause-specific mortality. JAMA Intern. Med..

[bb0305] Springmann M., Godfray H.C.J., Rayner M., Scarborough P. (2016). Analysis and valuation of the health and climate change cobenefits of dietary change. Proc. Natl. Acad. Sci..

[bb0310] Springmann M., Wiebe K., Mason-D'Croz D. (2018). Health and nutritional aspects of sustainable diet strategies and their association with environmental impacts: a global modelling analysis with country-level detail. Lancet Planet Health.

[bb0315] Springmann M., Spajic L., Clark M.A., Poore J., Herforth A., Webb P. (2020). The healthiness and sustainability of national and global food based dietary guidelines: modelling study. bmj.

[bb0320] The Vegan Society (2016). Find out how many vegans there are in Great Britain. https://www.vegansociety.com/whats-new/news/find-out-how-many-vegans-there-are-great-britain.

[bb0325] Theurl M.C., Lauk C., Kalt G., Mayer A., Kaltenegger K., Morais T.G. (2020). Food systems in a zero-deforestation world: dietary change is more important than intensification for climate targets in 2050. Sci. Total Environ..

[bb0330] Tilman D., Clark M. (2014). Global diets link environmental sustainability and human health. Nature.

[bb0335] Tziva M., Negro S.O., Kalfagianni A., Hekkert M.P. (2020). Understanding the protein transition: the rise of plant-based meat substitutes. Environ.Innov.Soc.Transit..

[bb0340] van Vliet S., Bain J.R., Muehlbauer M.J., Provenza F.D., Kronberg S.L., Pieper C.F. (2021). A metabolomics comparison of plant-based meat and grass-fed meat indicates large nutritional differences despite comparable nutrition facts panels. Sci. Rep..

[bb0345] Willett W., Rockström J., Loken B., Springmann M., Lang T., Vermeulen S. (2019). Food in the Anthropocene: the EAT–Lancet Commission on healthy diets from sustainable food systems. Lancet.

