# Supplementary material for: The role of plant-based alternative foods in sustainable and healthy food systems: Consumption trends in the UK
Source: Sci Total Environ. 2022 Feb 10;807:151041. doi: 10.1016/j.scitotenv.2021.151041 (PMC8724617; doi:10.1016/j.scitotenv.2021.151041)
Supplement: Supplementary file 1 — Supplementary material [file mmc1.docx]

Trends in UK Consumption of Plant-Based Alternative Foods

# Supplementary Information

## Contents:

1. List of items contained within each food group used in the analysis
2. Conversion factors used for calculating dietary energy intake from food items contained within composite dishes
3. Trends in mean daily consumption of selected food groups from 2008-20011 to 2017-2019, standardised for 2000 kcal daily energy intake
4. Mean daily consumption of selected food groups by category of plant-based alternative food consumer

## List of items contained within each food group; food number and subsidiary food group code as given in NDNS Nutrient Databank.

| Food Group | Food Item | Food Number | Subsidiary Food Group Code |
| --- | --- | --- | --- |
| Plant-Based Meat Alternatives | All items with subsidiary food group code 37K (meat alternatives) | | |
|  | Vegie burger purchased grilled | 4203 | 37L |
| Plant-Based Milks | Soya alternative to milk unsweetened | 650 | 13R |
|  | So good, fortified soya drink | 3410 | 13R |
|  | Soya alternative to milk sweetened calcium enriched | 8726 | 13R |
|  | Soya alternative to milk sweetened calcium, iron and vitamin enriched | 10974 | 13R |
|  | Soya alternative to milk sweetened plain | 8512 | 13R |
|  | Soya alternative to milk, fortified | 3769 | 13R |
|  | Soya alternative to milk, light, unsweetened, fortified | 10245 | 13R |
|  | Oat based milk alternative fortified | 10159 | 13R |
|  | Oat based milk alternative unfortified | 10966 | 13R |
|  | Rice dream alternative to milk, with added calcium | 10572 | 13R |
|  | Rice drink alternative to milk (not fortified) | 9494 | 13R |
|  | Hemp alternative to milk | 11241 | 13R |
|  | Coconut alternative to milk sweetened calcium enriched | 10836 | 13R |
|  | Almond alternative to milk fortified | 10898 | 13R |
|  | Almond alternative to milk unsweetened fortified | 11229 | 13R |
|  | Almond alternative to milk unsweetened fortified with calcium and vitamins | 11234 | 13R |
|  | Alpro rice alternative to milk original | 11150 | 13R |
| Other Plant-Based Dairy Alternatives | Soya cheese | 4082 | 14R |
|  | Tofu cheese | 9113 | 14R |
|  | Tofutti soya/tofu blend cheese products | 10539 | 14R |
|  | Pure soya soft and creamy dairy free spread | 10980 | 14R |
|  | Alpro plain soya yogurt fortified | 10575 | 15B |
|  | Yogurt, soya alternative, fruit, fortified with calcium only | 11145 | 15B |
|  | Yogurt, soya alternative, fruit, not fortified | 7743 | 15B |
|  | Yogurt, soya alternative, smooth fruit, fortified with ca, b2, b12, vit d and vit c | 9115 | 15B |
|  | Yogurt, soya alternative, with fruit pieces, fortified with ca, b2, b12 and vit d | 7127 | 15B |
|  | Pouring yogurt, soya alternative, vanilla, fortified | 11058 | 15B |
|  | Non dairy soya cream alternative | 10312 | 13B |
|  | Oat cream (non-dairy alternative) | 8231 | 13B |
|  | Cream, non-dairy, uht, aerosol | 6968 | 13B |
| Beans & Pulses | All items with subsidiary food group code 37I (beans and pulses) and 37C (baked beans) | | |
|  | All items listed within disaggregation category “beans and pulses” (variable name [beansg]), excluding items with subsidiary food group code 14R (other cheese), 37K (meat alternatives), 43R (sugar confectionery), and 53R (ice cream) | | |
| Nuts & Seeds | Items with subsidiary food group code 56R (nuts and seeds), excluding coconut products and quinoa | | |
|  | Honey nut shredded wheat, nestle | 6824 | 5R |
|  | Muesli, no added sugar with extra fruit & nuts | 6836 | 5R |
|  | Tracker bar peanut | 7966 | 7A |
|  | Tracker bar chocolate chip | 7967 | 7A |
|  | Cereal bars with nuts, no fruit, not coated, unfortified | 10058 | 7A |
|  | Cereal bars with fruit and nuts, not coated, unfortified | 10059 | 7A |
|  | Cereal bars with fruit and nuts, coated, unfortified | 10060 | 7A |
|  | Oshee vitamin muesli bar | 11327 | 7A |
| Vegetables | All items listed within disaggregation categories “tomatoes” (variable name [Tomatoesg]), “brassicaceae” ([Brassicaceaeg]), “yellow, red and dark green leafy vegetables” ([YellowRedGreeng]), and “other vegetables ([OtherVegg]), excluding items with subsidiary food group code 37I (beans and pulses), 37K (meat alternatives), 42R (crisps and savoury snacks), 43R (sugar confectionery), 8D & 8E (buns cakes and pastries) | | |
| Meat | All items listed within disaggregation categories “beef (red meat)” ([Beefg]), “lamb (red meat)” ([Lambg]), “pork (red meat)” ([Porkg]), “processed red meat” ([ProcessedRedMeatg]), “other red meat” ([OtherRedMeatg]), “burgers” ([Burgersg]), “sausages” ([Sausagesg]), “offal” ([Offalg]), “poultry (white meat)” ([Poultryg]), “processed poultry” ([ProcessedPoultryg]), and “game birds” ([GameBirdsg]), excluding items with subsidiary food group code 8D (manufactured buns cakes and pastries). | | |
| Milk | All items with subsidiary food group code 10R (whole milk), 11R (semi-skimmied milk), 12R (skimmed milk), and 60R (1% milk). | | |
|  | Milk goats summer | 623 | 13R |
|  | Milk goats winter | 624 | 13R |
|  | Goats milk uht | 3237 | 13R |
|  | Semi-skimmed goats milk, pasteurised | 10397 | 13R |
|  | Skimmed goats milk pasteurised | 10416 | 13R |
|  | Milk sheeps summer | 625 | 13R |
|  | Milk sheeps winter | 626 | 13R |
|  | Lactose free semi skimmed milk | 9493 | 13R |
|  | Lactose free whole milk | 10932 | 13R |
|  | Lactofree fresh skimmed milk fortified | 11180 | 13R |
|  | Buffalo milk | 10122 | 13R |
| Other Dairy Products | All items listed within disaggregation categories “cottage cheese” ([CottageCheeseg]), “cheddar cheese” ([CheddarCheeseg]), and “other cheese” ([OtherCheeseg]), excluding plant-based alternatives, and excluding items with subsidiary food group code 2R (white bread), 7A & 7B (biscuits), 8D & 8E (buns cakes and pastries), 9G & 9H (cereal based puddings), 15C & 15D (dairy desserts). | | |
|  | All items with subsidiary food group code 13B (cream) and 15B (yogurt), excluding plant-based alternatives. | | |

## Conversion factors used for calculating dietary energy intake from food items contained within composite dishes.

Where composite dishes were coded as single entries within the NDNS dataset and the food item components of the dish were known, grams of intake were converted to kcal using the energy values per gram of the constituent food items obtained from the nutrient databank. Many of the composite dishes contained more than one food item from a food group, or the primary ingredients were not able to be identified from the name of the dish, in which case grams of intake were converted to kcal using a value derived from the average energy per gram of all listed individual food items of the respective food group contained within the nutrient databank.

| Food Group | Energy Conversion Factor (kcal/gram) |
| --- | --- |
| Beans & Pulses | 0.91 |
| Nuts & Seeds | 5.99 (nuts) |
| Vegetables | 0.347 |
| Meat | 2.15 |
| Other Dairy Products | - 1. (yogurt), 3.24 (cheese) |

## Trends in mean daily consumption of selected food groups from 2008-20011 to 2017-2019, in grams/capita/day, standardised for 2000 kcal daily energy intake; aggregate change over time.

|  | 2008-2011 | 2011-2014 | 2014-2017 | 2017-2019 | *p* test for trend |
| --- | --- | --- | --- | --- | --- |
| Beans & Pulses | 15.6 (14.3 - 16.8) | 16 (14.7 - 17.3) | 17.3 (15.5 - 19.0) | 16.2 (14.7 - 17.7) | 0.238 |
| Nuts & Seeds | 2.1 (1.8 - 2.4) | 3.0 (2.7 - 3.4) | 4.1 (3.6 - 4.7) | 4.2 (3.6 - 4.8) | <0.01 |
| Vegetables | 164.5 (159.5 - 169.4) | 156.9 (152.4 - 161.3) | 154.5 (149.7 - 159.3) | 156.8 (150.7 - 162.9) | 0.02 |
| Meat | 122.2 (119.0 - 125.3) | 115.5 (112.3 - 118.7) | 113.5 (110.0 - 117.0) | 102.8 (98.9 - 106.8) | <0.01 |
| Milk* | 216 (208.3 - 223.6) | 217.4 (210.3 - 224.5) | 208.4 (199.9 - 216.9) | 197.7 (188.3 - 207.1) | <0.01 |
| Other Dairy Products* | 51.2 (48.9 - 53.5) | 51 (48.7 - 53.2) | 49.1 (46.6 - 51.5) | 50.9 (48.0 - 53.8) | 0.515 |

* Excludes dairy products used in baked goods, confectionary and desserts, and yogurt, cream and milk used in composite dishes.

Brackets denote 95% confidence interval.

## Mean daily consumption of selected food groups by category of plant-based alternative food consumer (defined by tertiles of consumption), in grams/capita/day.

##
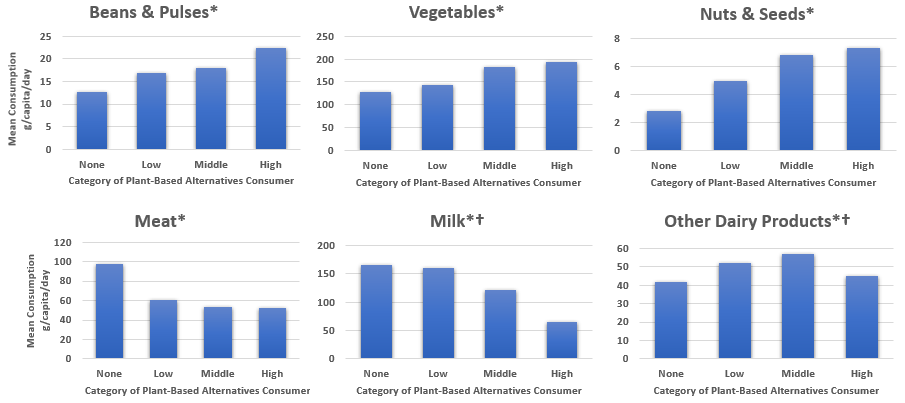


*indicates *p* value <0.01. †Excludes dairy products used in baked goods, confectionery and desserts, and yogurt, cream and milk used in composite dishes.
